# Bio-Fabrication: Convergence of 3D Bioprinting and Nano-Biomaterials in Tissue Engineering and Regenerative Medicine

**DOI:** 10.3389/fbioe.2020.00326

**Published:** 2020-04-16

**Authors:** Nicola Di Marzio, David Eglin, Tiziano Serra, Lorenzo Moroni

**Affiliations:** ^1^AO Research Institute, Davos, Switzerland; ^2^Department of Health Sciences, Università del Piemonte Orientale (UPO), Novara, Italy; ^3^Institute for Technology-Inspired Regenerative Medicine, Maastricht University, Maastricht, Netherlands

**Keywords:** biofabrication, tissue engineering, bioprinting, nano-technology, regenerative medicine

## Abstract

3D Bioprinting (3DBP) technologies open many possibilities for the generation of highly complex cellularized constructs. Nano-biomaterials have been largely used in tissue engineering and regenerative medicine (TERM) for different purposes and functions depending on their intrinsic properties and how they have been presented in the biologic environment. Combination of bioprinting and nano-biomaterials paves the way for unexpected opportunities in the biofabrication scenario, by improving critical weakness of these manufacturing processes while enhancing their efficiency by spatially arranging nano-features. 3D organization of cells is fundamental for a successful design and maturation of native tissues. A critical challenge for the production of biological constructs is to support and guide cell growth toward their natural microenvironment, ensuring a harmonious presence of specific biochemical and biophysical cues to direct cell behavior. Also, precise arrays of stimuli need to be designed to induce stem cell differentiation toward specific tissues. Introducing nano-sized bioactive material can direct cell fate, playing a role in the differentiation process and leading to the biofabrication of functional structures. Nano-composite bio-ink can be used to generate cell instructive scaffolds or either directly printed with cells. In addition, the presence of nano-particles within 3D printed constructs can lead to control them through multiple external physical stimuli, representing an additional tool for healthcare applications. Finally, there is an emerging interest to create biological constructs having active properties, such as sensing, motion or shape modification. In this review, we highlight how introducing nano-biomaterials in bioprinting approaches leads to promising strategies for tissue regeneration.

## Introduction

Bioprinting is the automated fabrication of 3D cellularized constructs for the generation of advanced *in vitro* models and therapeutics. This is usually based on a layer-by-layer approach with the deposition of biomaterials in a precise spatial arrangement following a computer aid design (CAD) model. When the printed material is comprised of biological molecules or cells, it is called bio-ink and is usually made of a hydrogel (Moroni et al., 2018). The bioprinted model may be directly maturated into a functional tissue or the biofabricated 3D construct is used to create a template where cells can be seeded or grown into. In the last decades, several 3DBP have been developed. Recently a new classification of the biofabrication techniques has been pointed out in the review work of Moroni et al. where the biofabrication techniques have been mainly grouped in bioprinting and bioassembly. It encompasses instructive scaffolds able to steer cell activity through engineering surface or hierarchical properties. Spatial resolution/time for manufacturing (RTM) ratio has been also introduced as a way to quantitively classify the existing technologies based on the efficiency of the fabrication method. Following this classification, the technologies with higher RTM ratio have higher efficiency process. Higher delivery rate leads to have low resolution, on the other hand increasing details resolution leads to have longer manufacturing time (Moroni et al., 2018). Each of these technologies have benefits and disadvantages related to the inherent technical processes used: jetting, extrusion, sintering to deposit material into layers. Therefore, the choice among them is based on the final application, the complexity of the model, the envisioned bio-ink properties, the time needed for the fabrication and available economical investment. For example, piezoelectric or thermal actuators used in the inkjet bioprinting, which dispenses the bio-ink drops, could affect cell viability. Instead, in laser assisted techniques, no mechanical stress is applied to the cells, but the collateral effects of cells exposure to the laser beam are not completely understood yet (Mandrycky et al., 2016). Extrusion bioprinting generates a mechanical stress on the bio-ink which is considered a possible risk for cell viability. Therefore, bioprinting parameters, like speed and pressure, as well as nozzle’s shape and diameter must be carefully optimized together with the bio-ink properties. Overall, high resolution in cells deposition and distribution, nozzle clogging, and 3D functional engineered tissue fabrication remain the main open issues currently under research (Mandrycky et al., 2016).

Nano-biomaterials have less than 100 nm in dimension (Yang et al., 2011), and due to their size, they can interact and be up-taken by cells inducing cellular response. Nano-biomaterials have the ability to stimulate cell’s receptors and be instructive for the cell to adopt specific behaviors (An et al., 2013), this is why they have been used also in TERM. Reported nano-biomaterials have been made of metals, polymers, and ceramics such as bioglasses, therefore including the whole spectrum of materials. Yang et al. (2011) classified them based on their use in soft or hard tissue engineering (TE) applications. For example, in cardiac TE the functions of endothelial and smooth muscle cells have been improved by different formulation of nano-biomaterials as reviewed by Zhang and Webster (Zhang and Webster, 2009). Nano-structured materials have shown to play an active role in the biologic environment and have been used to enhance biomaterial properties. The following examples resume their most relevant applications in the biologic/healthcare scenario. Surfaces functionalization of biomedical devices with nano-biomaterials have been proved to solve several problematics. Titanium nano-formulation reduced the inflammation and thrombus creation on vascular stents (Choudhary et al., 2006). Poly(ether urethane)-based nano-structured poly(lactic-co-glycolic acid) biofilm for cell growth promoted cell adhesion and functionality of bladder smooth muscle cells (Thapa et al., 2003). Silver nano-particles used for catheters functionalization decreased the risk of infection (Roe et al., 2008). Titanium nano-rough surfaces revealed to have antibacterial adhesion effect (Puckett et al., 2010). The nano-scale size allows these materials to be active at the cellular and sub cellular levels, therefore they have been used also for instruct and guide cells, for example CNT array configuration modified osteoblasts orientation (Giannona et al., 2007). Moreover, the hierarchical structure of micro- and nano-environments of biologic tissues can be mimic by use of nano-biomaterials. Typically, the nano hydroxyapatite (nHA) and tricalcium phosphate nano-composite (nTCP) have shown the ability to be manufactured into bone’s hierarchical like structure, with relevant microenvironment and mechanical properties (Yang et al., 2011). Furthermore, nano-biomaterials interaction directly with the cell or from the cell’s internal environment, made them perfect candidate for local drug delivery. Sustained release lowers the systemic drug dose and aims for direct targeting. Specifically, polymer nano-particles (NPs) can be tuned for sustaining release of drugs in a controlled manner (McCarron et al., 2008), but also ceramic NPs, which present complementary physical properties have been used as well in drug delivery (Yang et al., 2010). Additionally, nano-biomaterials can be remotely controlled and stimulate with different energy sources such as near-infrared, ultrasound and magnetic fields (Marino et al., 2018). Therefore, they can act as nano-transducers at the cellular or subcellular levels allowing for remote modulation of cells functions as biochemical signaling pathways (Bonnemay et al., 2015), gene/protein expression (Stanley et al., 2012), electrochemical reactions (Xu et al., 2017), neural firing (Yong et al., 2014), and muscle contraction (Marino et al., 2017).

One of the most commonly used nano-biomaterials in tissue engineering are carbon nano-tubes (CNTs). They have a cylindrical structure made of rolled graphene sheets characterized by sp2-hybridized carbon atoms bounded in hexagonal configuration. CNTs can be realized in a single or multiple concentric rolled graphene layers. In the latter case, they are named multi-walled CNTs (MWCNTs). Their dimensions are usually up to 100 nm in diameter and in the micrometers range in length (Ménard-Moyon, 2018). Their high aspect ratio is important for their interaction with cells. CNT’s surface can be functionalized to increase even more their biocompatibility and exploit them for their outstanding electrical properties in neural TE for a faster healing of damaged nerves. Proving this for the first time, Mattson et al. (2000) published the feasibility of growing embryonic rat-brain neurons on MWCNTs. They observed that when neurons were grown onto unmodified MWCNTs only few neurites extended from the neurons, while when they were grown onto nano-tubes functionalized with the biomolecule 4-hydroxynoneal, they developed several neurites with multiple branches (Mattson et al., 2000).

**FIGURE 1:**
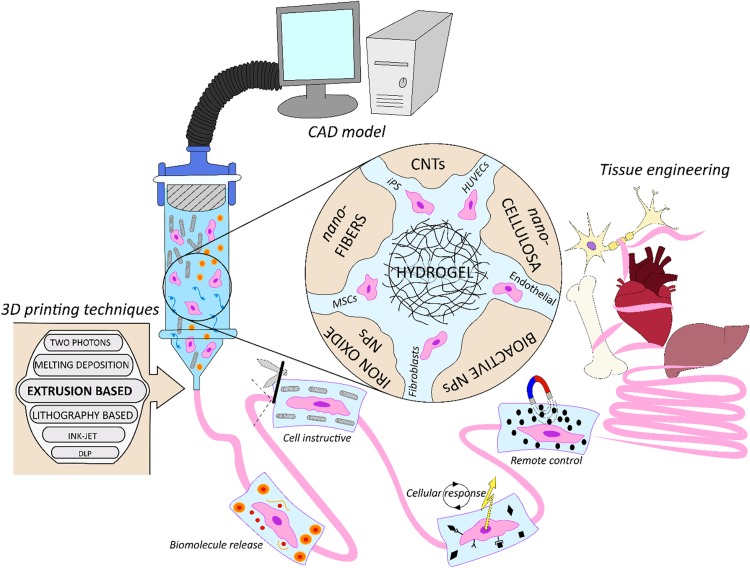
Schematic representation of nano-composite bio-inks functionalities in biofabrication for tissue engineering. From the computer assisted design (CAD) model toward the artificial organ or scaffold realization, exploiting multiple biofabrication techniques.

In Figure 1 we schematically represent the focus of this work. We review how 3DBP have been used in synergy with nano-technology for applications in TERM, analyzing what are the benefits to combine them for biofabrication strategies. Initially we discuss the cell-laden nano-composite bio-inks used in 3DBP, focusing on the applications of biomolecule release, biomimicking of the extracellular matrix structure, triggering cell response and cell instructive remote control. Next, cell-free nano-composite bio-inks for 3DBP are discussed. Finally, new technological applications in bionic tissues and soft robotics for TERM are introduced.

## Cell-Laden Nano-Composite Bio-Inks

Currently available bio-ink formulations are limited due to the requirements of “good” printability (e.g., shear thinning, shape retention). Moreover, high cell viability and long-term function are necessary. Considering the extrusion based 3DBP for instance, Pluronic hydrogels have great printability properties, but cells do not survive and proliferate in the long-term *in vitro* culture in a Pluronic concentration higher than 5 w:v% (Khattak et al., 2005; Fedorovich et al., 2009). A possible solution was found by nano-structuring these hydrogels to increase their biocompatibility. Pure Pluronic (PF127), combined with diacrylated Pluronic F127 (PF127-DA), creates nano-pores after wash-out of the pure PF127 from the UV-crosslinked structure. Muller et al. combined the new hydrogel formulation with bovine chondrocytes, bioprinted them in a simple structure and cultivated them up to 14 days. Cell viability at the end of the experiment was significantly higher compared to their viability in the bulk Pluronic gel structure, reported to be 86.3% compared to 61.6% (Müller et al., 2015). Complementary to the creation of nano-pores inside a hydrogel, embedding nano-biomaterials inside the bio-ink allowed for nano-composite bio-inks formulation directed to specific achievements in cellular constructs for TERM here reported.

### Nano-Composite Bio-Inks for Biomolecule Release

**FIGURE 2:**
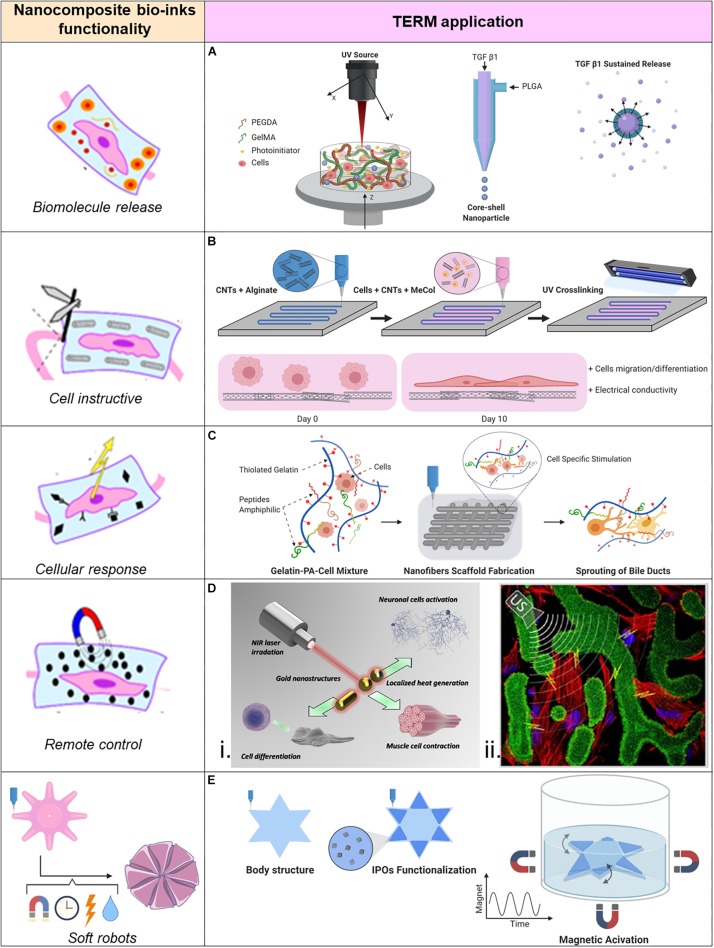
Applications of nano-composite bio-inks in tissue engineering and regenerative medicine (TERM). **(A)** 3D bioprinting of mesenchymal stem cell-laden construct with core-shell nano-spheres for cartilage tissue engineering. **(B)** UV-Assisted 3D bioprinting of nano-reinforced hybrid cardiac patch for myocardial tissue engineering. **(C)** Tailoring nano-structure and bioactivity of 3D-printable hydrogels with self-assemble peptides amphiphile (PA) for promoting bile duct formation. **(Di)** Smart inorganic nano-particles for wireless cell stimulation, **(ii)** Two-Photon lithography of 3D nano-composite piezoelectric scaffolds for cell stimulation. **(E)** Stimuli-responsive nano-composite for 3D magnetic soft robots.

Chemical cues are important for stem cell differentiation, and specific stimuli are particularly necessary in order to induce differentiation in bone or cartilage tissues. Growth factors are commonly provided in tissue engineering because they are responsible for spatial and temporal cell functions. Unfortunately, without protecting these molecules, they experience degradation and quick elimination even though they are administrated in high dose (Chen et al., 2010). This is even more critical when applied within a bio-ink, which process may further decrease stability of fragile proteins. A novel approach introduced by Zhu et al. (Figure 2A) allows for nano-carriers of transforming growth factor beta 1 (TGF-β1) directly inside the bioprinted cartilage construct, from which TGF-β1 can be sustained released, obtaining a significant improved MSCs chondrogenesis differentiation. In this study stereolithography based 3DBP was used to create the cartilage construct. A movable head with a UV-source was used to crosslink the square shaped cartilage construct. The bioink was a gelatin methacrylate (GelMA)-based hydrogel combined with TGF-β1 loaded nano-spheres (average size of 120 nm) which where fabricate through a co-axial electrospraying method (Zhu et al., 2015). The nano-carrier’s surrounding shell of poly(lactic-co-glycolic acid) (PLGA) slowly degrades and sustains the release of its content (Danhier et al., 2012). The qPCR analysis revealed that the expression levels of collagen II and aggrecan of MSCs laden into the hydrogel increased when the bioink was enriched with TGF-β1 nano-carriers (Zhu et al., 2018). This is a valid strategy for embedding biomolecules into bioprinted constructs. Indeed, with this approach is possible to recapitulate the dynamic presence of biomolecules into the ECM. However, tuning the biomolecule release could require a long optimization time to achieve the desired release profile. This top-down fabrication approach, though, leaves back a not neglectable amount of unused material, which is not optimal if the material is precious or its availability is limited. Envisioning clinical applications, the donor biological material is often a limiting factor. Moreover, the fabrication time could be reduced if the UV-light exposure could be volumetric instead of punctual as with a UV-light laser source.

### Cell Instructive Nano-Composite Bio-Inks for Matrix Properties Enhancement

Embedding nano-biomaterials inside bio-inks can help to drive the cell behavior via modulation of mechanical and structural properties of cell’s microenvironment. Electrospinning have been also used to deposit layers of random or aligned nano-fibers hierarchically integrated in a 3D multidimensional structure. This manufacturing concept was used by Yeo et al. (2016) who created a hierarchical supporting structure based on 3D printing. Onto a main layer in poly(ε-caprolactone) (PCL) was electrospun a layer of micro/nano PCL fibers in either random or organized way. Another layer of bioprinted myoblasts was deposited on top of these layers, and the whole 3D structure was at the end rolled in a cylindric fashion. From the cell-laden layer, the myoblasts could migrate and populate the scaffolds following the aligned electrospun nano-fibers (Yeo et al., 2016). Although this approach seems to be promising to instruct myoblast cells to follow a certain orientation inside the engineered tissue, it requires consecutive application of different technologies. Also, it does not allow for controlled spatial organization of the electrospun fibers. Instead, nano-features can be loaded directly into the bio-ink, which can be directly 3D organized as designed, with the purpose of cell polarization and support cell growth.

#### High Fibrillar ECM Based Tissues

Cellulose nano-fibrils (NFC) were recently used due to their biocompatibility to create a nano-fibrillar bioink (Backdahl et al., 2006). It has been shown that it is possible to embed NFCs into commonly used hydrogels as alginate (SLG100) with a shear viscosity close to zero (tested composition of 2–4% w/w), the selected bio-ink formulation resulted 80:20 NFC/alginate (w/w) with 2% w/w of NFC, 0.5% w/w of alginate and 97.5% of water content. This formulation was identified as optimal because of the good gelling while crosslinking and a good shape fidelity, therefore used as a bio-ink to deposit human chondrocytes (Markstedt et al., 2015). The synergy of alginate and NFC revealed functional, because the bioprinted structures with low viscosity alginates alone resulted in poor shape fidelity, the viscosity was indeed too low to keep the shape fidelity. When NFC was included, the resulting bioink had the rheological properties of NFC and the cross-linking capabilities of alginate. With this technique Markstedt et al. bioprinted complex cartilage tissues like ear and meniscus disk. The results showed that this novel bio-ink formulation is suitable for cell culture and tissue engineering, possibly due to the collagen ECM-like structure reproduced by the dispersed cellulose nano-fibers. Following their study, Nguyen et al. bioprinted for the first time human derived induce pluripotent stem cells (iPSCs), they tested NFC-alginate (NFC/A) and NFC-hyaluronic acid (NFC/HA) as bio-ink’s matrix. In both the formulations the NFC reproduced the collagen matrix and it sustained cell attachment. The authors concluded that NFC/A bio-ink formulation was the most suitable for iPSCs bioprinting to support cartilage production. Indeed, in the presence of NFC/HA, iPSCs showed low proliferation and immediately phenotipic changes. Instead, with NFC/A, iPSCs proliferated for 5 weeks while remaining pluripotent, but then they observed hyaline-like cartilagineus tissue with expression of collagene type II (Nguyen et al., 2017). The mentioned results suggest that the introduction of natural nano-fibers into well-known hydrogels is a valid strategy to create a suitable matrix for engineering tissues, which are characterized by a high component of fibrillar ECM. Nano-fibrillar enrichment of plain hydrogels infact, gives to the cells a local reference, which they can sense and use as 3D support to proliferate.

#### Low Fibrillar ECM Based Tissues

For the muscular tissue instead, the ECM does not play a bulk role as in cartilage or bone tissues. Therefore, the engineering process of this tissue has to face different requirements. Conductivity is essential for the propagation of the electric signal, which activates this tissue to contract. The cardiac tissue physiology is strictly connected with electrical potential signals generated to coordinate the cardiomicyte beating. Therefore, TE of functional cardiac tissue focuses closely on the electrical conduction of the manufactured tissue. CNTs are well known for their electrical and mechanical properties, hence useful for cardiac TE. A functional cardiac patch for myocardium infarct treatment (Figure 2B) was developed in a study performed by Izadifar et al. In this study CNTs have been functionalized with carboxyl groups to chemically bind to the alginate matrix after alginate crosslinking with calcium chloride. The nano-compound bio-ink was used as reinforcement and conductive construct for a bioprinted cell-laden methacrylated collagen hydrogel. In this study, human coronary cardiac endothelial cells (HCAECs) were bioprinted to create a biologically relevant cardiac patch. The CNTs together with alginate were able to create a nano-fibers network, with characteristic dimension of ∼25–100 nm inside the patch, which was able to significantly reduce the patch’s impedance at the physiologic frequency of 5 Hz. With the synergistic effect of CNTs reinforcement in the bioprinted patch, enhanced HCAECs proliferation, migration, and differentiation was obtained. Moreover, the cells were observed to be organized in a lumen like configuration inside the patch along the 10 days of the *in vitro* study (Izadifar et al., 2018). In addition to the increasing conductivity, CNTs can be fabricated with multiple concentric walls (MWCNT) and can further increase the mechanical properties of the bioprinted structure. In a study carried out by Zhang et al. MWCNTs were introduced to the bioprinted structure as reinforcement of the fabricated vascular structure in order to mimic the mechanical properties of the natural blood vessels. An extrusion-based system with a coaxial needle was designed for bioprinting an artificial vascular conduct. The bioink was made of a 4% (w/v) alginate solution enriched with 1% (w/v) MWCNTs into which human umbilical vein smooth muscle cells were resuspended and was compared to a bio-ink formulation of 4% alginate alone. The introduction of 1% MWCNTs into the bioink affected the dimensions of the artificial vessel conduit in terms of wall thickness, vessel diameter, and lumen diameter which were statistically significantly bigger compared to the bioprinted conduit without MWCNTs or with 0.5% (w/v) MWCNTs. Moreover, the tensile strength increased in the 1% nano-composite vessel conduit (422 ± 23 kPa compared to 382 ± 19 kPa for the control) and the cell viability was not affected from the presence of MWCNTs (Zhang et al., 2014). Even though the results of CNTs bioink functionalization are promising, there are still open questions about their safety when they are used in clinical applications. More research is necessary to clarify *in vivo* side effects of CNTs.

## Cellular Response Stimulated by Bioactive Nano-Composite Bio-Inks

The introduction of nano-sized bioactive material can stimulate cells to behave differently, playing a role in the differentiation process and leading to the biofabrication of bio-functional structures. Signaling molecules must be sensed from the cells in order to have high cell survival rates and instruct them to produce specific proteins or explicit specific functions. For example, functional peptides or particular proteins can induce cell adhesion (Khoo et al., 2015). These elements are naturally present in the ECM. Example of functional peptides is the laminin-derived peptide sequence IKVAV (Ile-Lys-Val-Ala-Val), which belong to the peptide amphiphiles (PAs) family. PAs are molecules with the capability of self-assembly into nano-fibers (Hartgerink et al., 2001; Hendricks et al., 2017) and have been used in different TE applications (Silva et al., 2004; Tysseling-Mattiace et al., 2008; Webber et al., 2011). Yan et al. combined these nano-fibers into a bio-ink for bioprinting of a cell-laden liver construct (Figure 2C). IKVAV PAs nano-fibers have been combined with a matrix of thiolated gelatin where cholangiocytes were suspended and used as a bio-ink to fabricate the liver-like biological construct, which was then kept in culture for up to 14 days. Within the nano-functionalized construct, cholangiocyte cysts developed branching tubular structures which grew over time. In the plain gelatin cell-laden scaffold, cholangiocyte cysts didn’t form any branches, although they grew in size. Moreover, the bile ducts were proven to be mature and functional, as a fluorescent bile acid analog was transported inside the central lumen and inside the cells (Yan et al., 2018). This study is a clear evidence of how a plain matrix like gelMA is often not enough to mimic the right environment for the cells to mature in functional biological structures. It also supports the developing process of composite hydrogel; indeed the reward for a more physiologically relevant matrix is clearly appreciated.

In neural tissue regeneration instead, a graphene-based nano-biomaterial formulation has been used to differentiate neural stem cells in neurons (Park et al., 2011) and stimulate neural network formation (Serrano et al., 2014). Graphene nano-platelets were combined with gelatin methacrylamide (GelMA). This bio-ink (G-GelMA) with a concentration of 1 mg/mL of graphene was used to bioprint neural stem cells-laden scaffolds by stereolithography. At 14 days, neural differentiation was assessed by neurites imaging, β-tubulin III resulted high expressed in contrast to glial fibrillary acidic protein (GFAP). Graphene nano-platelets embedded within the bioink did not show cytotoxicity, instead they demonstrate an increased biofunctionality of the printed compound and it supports the need for further research in this direction (Zhu et al., 2016).

In bone tissue engineering, bioglass (BG) nano-formulation have been significantly employed in biofabrication. Nano-BG are bioactive materials of several compositions (Hench, 2011; Jones, 2013). These nano-biomaterials have been used in bioprinting process (Luo et al., 2013), because of their mainly osteogenic properties which were reviewed in 2013 (Jones, 2013). Traditionally, cells have been grown onto BG bulk structures, but now BG nano-particles have started to be included in bio-inks formulation and they maintain their osteogenic properties. Wang et al. (2014) combined BG nano-particles with a characteristic size of 55 nm in an alginate/gelatin hydrogel with bone-related SaOS-2 cells laden. The tested BG formulation was 100 mMoles/L polyPNCa^2+^-complex and 50 mMoles/L ortho- silicate (silica) or 50 mmoles/L biosilica. Results showed that SaOS-2 cells proliferated more and demonstrated an increased mineralization when the BG nano-formulations was added to the bioink (Wang et al., 2014). Not only BG, but also bioactive ceramic nano-particles such as nano-hydroxyapatite (nHA) are able to stimulate osteogenesis as proved by bioprinted hMSCs-laden photopolymerizable hydrogel constructs combined with bioactive ceramic nano-particles (Cui and Boland, 2009; Cui et al., 2012). A later study wanted to assess the differences between nHA and BG in terms of osteogenic properties. Hence, a hMSCs-laden poly(ethylene glycol) dimethacrylate hydrogel was enriched with nHA or BG nano-particles and bioprinted. After 21 days, nHA induced faster osteogenesis in hMSCs than BG nano-particles loaded bio-ink. Cell viability was also reported to be higher when hMSCs were co-printed with nHA in comparison to BG (Gao et al., 2014). Song et al. (2016) supported the osteogenic capabilities of nHA-composite bio-ink, when they studied the influence of nHA as differentiation promoter for human adipose-derived mesenchymal stem cells (hASCs). In this study, the cell-sodium alginate-gelatin mixture was enriched with 10 g/L nHA and the cells were grown for 14 days, after which the gene expression was evaluated by PCR analysis. Here it was shown that osteogenesis-related genes (RUNX2, osterix, and osteocalcin) significantly increased their expression when compared to the bioprinted samples without nHA (Song et al., 2016). The lack of reported toxicity and no reduction of cell proliferation suggests that nHA is a valid added component for a bioink with envisioned application in bone tissue engineering.

### Cell Instructive Strategies via Remote Controlled Nano-Composite Bio-Inks

Nano-composite bio-inks can be designed to actively interact with the cells hosted inside the bioprinted structure. Indeed, the possibility to control nano-particles with multiple external physical stimuli introduces a complementary tool in combination with 3DBP. Superparamagnetic nano-particles (NPs) can be organized and manipulated by introduction of an external magnetic field, which allow for further patterning capabilities in addition to the initial pattern obtained by 3DBP. By combining these technologies, it is indeed possible to locally modify a bioprinted structure as showed in the work of Buyukhatipoglu et al. They investigated how incorporation of iron oxide NPs into alginate solutions influences the bioprinting parameters and cell viability. Manufacturing parameters did not change when the nano-functionalized bioink or the non-enriched one were bioprinted. Iron oxide NPs did not affect cell viability especially if they were loaded in low concentration inside the hydrogel rather than when they let the NPs to be uptake by the cells. After bioprinting they were able to move and aggregate the NPs with a magnet showing how hydrogel’s viscosity hardly influences the nano-particle manipulation after bioprinting (Buyukhatipoglu et al., 2009, 2010). They claim as application of this synergistic technologies the possibility to move from one site to the other biological materials inside the bioprinted construct when needed. Although it seems a promising approach due to reported ability to move cells after they uptake NPs, moving NPs across the construct could affect the cell viability. Moreover, the printed shape’s fidelity could change after magnetic manipulation. Ultimately, the drag force of the magnetic field could also displace not targeted biomaterials on the way.

In addition to the magnetic manipulation, external ultrasounds (US) can be used to remotely control piezoelectric nano-particles (piezo-NPs). Indeed, following the mechanical deformation induced by ultrasounds stimulation, piezo-NPs produce electricity. Therefore, by including them into bioinks it is possible to manufacture biological structures with piezoelectric elements at the nano-dimension. It was proven that mentioned nano-biomaterials can stimulate cells (Guo et al., 2012; Lee and Arinzeh, 2012; Royo-Gascon et al., 2013). This approach was used to bioprint a biologic construct for contactless cell stimulation (Figure 2Dii) using two-photon lithography and commercial photoresist, Ormocomp, doped with barium titanate piezo-NPs (10 wt%). “Osteo-Prints” is the name of the 3D construct inspired by trabecular bone geometry and it is known to induce osteogenesis (Marino et al., 2014). It was later functionalized with piezo-NPs and the osteogenesis induction on hSaOS-2 cells was studied. The topological and electric stimuli resulted to enhance differentiation of hSaOS-2 cells. In fact, the comparison of the established osteo-inductive behavior of “Osteo-Prints” with the same construct enriched with piezo-NPs combined with US stimuli showed significantly increase of collagen type I (key protein of osteogenesis) and significantly decrease of Ki-67 (key factor of cell proliferation phases) expressions (Marino et al., 2015).

## Cell-Free Nano-Composite Bio-Inks

Usually referred as scaffolds, the 3D constructs, which will be populated in a second moment from cells can also be realized with a nano-composite bioink for several advantages. First, cells do not undergo the stressful fabrication processes as the shear stress experienced during extrusion from a needle. Moreover, the spectrum of fabrication technologies available for the manufacturing is wider due to absence of live matter involved in the process. Also, the scaffold’s applications are more oriented toward regenerative medicine as tissue gap filling, which will be later populated by surrounding tissue’s cells. Lastly, it could be cheaper to introduce them in clinical applications because they can be manufactured without patient’s biological material, which instead would increase significantly the production costs. In recent years the above discussed cellulose nano-fibrils (CNFs) were also used to create cell-free scaffolds with a greener approach as reported by Xu et al. They were inspired by the cell’s wall structure in plants, which present a strong and flexible interface due to the affinity between heteropolyssaccharides and cellulose nano-fibrils (Launey and Ritchie, 2009). Hemicellulose is an alternative material for hydrogel nano-structuring because, compared to other nano-biomaterials, it is cheaper to produce as it is economically fractionable from wood sources. Moreover, hemicellulose is widely present in nature and is non-toxic, biocompatible and biodegradable. Therefore, they created an ink formulation of CNFs combined with a UV-crosslinkable hemicellulose derivate which was used in a bioprinting extrusion technique to realize light weight scaffolds and complex structures with high resolution and good shape fidelity (Xu et al., 2019).

Recreate the collagen’s nano-fibers organization inside the ECM is an old and still open challenge. In the past, thermally induced phase separation (TIPS) has been used. TIPS exploits the quenching phenomena of polymer solutions (Ma and Zhang, 1999; Holzwarth and Ma, 2011). Through this technique, therefore, it was possible to create domains in the gel’s structure with higher polymer concentration which could be fixed in place by crystallization, gelation, and vitrification (van de Witte et al., 1996). Initially, this could be achieved by first fabricating a 3D sacrificial mold and after, casting the polymer solution. By applying TIPS technique, the sacrificial mold was eliminated and the desired architecture of polymeric nano-fibers could be obtained as negative shape of the mold (Chen et al., 2006; Akbarzadeh et al., 2015). A recent approach combined extrusion-based additive manufacturing with TIPS and allowed for the direct fabrication of scaffolds made of poly(L-lactide) (PLLA) with nano-fibrous strands without a sacrificial mold. With this approach a self-supporting structure was manufactured at room temperature, which presented characteristic dimensions from the nano- to the centimeter-size. The scaffold pores’ size is critical for tissue regeneration. In this study, they succeeded to have nano-fibers diameters around 180 nm into the printed strands without additional material added to the ink, only relying on the self-assemble capabilities generated by the fabrication process. The scaffold’s nano-fibrous structure enhanced proteins adsorption, cells adhesion and MSCs chondrogenic differentiation compared to scaffolds with smooth surface. Furthermore, with the introduction of nHA into the nano-fibers, the mechanical properties were further improved and the seeded MSCs were differentiated into an osteogenic lineage (Prasopthum et al., 2018).

The ECM has a complex 3D architecture which not only presents nano-scale features, but it also presents a chiral nature of the bioactive molecules contained. It was shown that, biomolecules and their enantiomers can drive cells affinity for nano-composites present inside the hydrogels (Kehr, 2016). In a recent study, a triphasic chiral nano-composite hydrogel for bioprinting was used to investigate how cells could distinguish between different chiral surfaces, and how they could selectively populate the scaffold which was functionalized with the most preferred biomolecule enantiomer. Motealleh et al. (2019) created a nano-composite bioink, embedding silica-based nano-particles, which were functionalized with bioactive molecule enantiomers within alginate hydrogel. A multifunctional structure composed of three scaffolds was bioprinted, two of them loaded with nano-particles which exposed opposite enantiomers of a biomolecule. Using this configuration, the authors studied how cells from the third cell-laden scaffold, could populate and migrate toward one or the other scaffold following the presence of biomolecule enantiomer for which the cells had more affinity (Motealleh et al., 2019). Following these promising results, nano-composite bio-ink functionalized with specific enantiomers are suitable material for 3D bioprinted constructs used for selectively attract the cells of the natural tissue to be regenerated.

## Nano-Composite Bio-Inks for Bionic Tissues and Soft Robots

Exploring the synergistic combination of 3DBP and nano-technology, fabrication of complex biologic structures with active properties is the futuristic application of such technologies. Sensing, moving capabilities and shape modification have been included in biologic constructs. Functional electronic components and biological tissue have been integrated through bioprinting of nano-enriched bio-ink. For example, nano-electronics materials and cell-laden hydrogels have been deposited together in a model of a human hear. With this knowhow, it is possible to manufacture cyborg tissues which are three-dimensional combination of electronics and engineered tissue. In the example of the bionic hear, the bioprinted construct could receive and transmit radiofrequency sounds. This proof of concept showed the integration of electronic circuits built up with nano-elements in a 3D biological construct. It overtakes the planar flexible electronic devices and sensors previously used, even though now the conductive elements are discrete and not continuous (Mannoor et al., 2013).

A bionic tissue must mimic the native organ’s functionality, replacing a lost body function or even improving it. Designed nano-biomaterials surfaces combined with additive manufacturing have shown potential in engineering bioactive devices. For example, natural red blood cell (RBC) membrane have been wrapped onto PLGA nano-particles (RGB-NPs) to mimic natural RBCs with a final dimension of 133 nm in size. Exploiting the natural behavior of RBC membrane, RBC-NPs have the capacity to bind, through a non-specific bonding, hemolytic toxins. In doing so, they can be integrated as an active component in a detoxification bioprinted device, which was developed by embedding RBC-NPs inside a hydrogel for biomimetic detoxification properties close to the liver function. The authors of this study stated that with their approach they demonstrated a functional solution for localized patient specific medicine applications (Chen et al., 2017). The efficacy of the device in a dynamic flow configuration was not proved and this is a current limitation of their work. Indeed, the device was only tested in a static experimental setup where it was used to detoxify a solution in which it was immerged.

Tissues and organs of the human body have motion capability which influences their regeneration and physiology. Therefore, actuators integrated through biofabrication process into the biological constructs could recapitulate the mobility capacities of the native tissue. The ability to change over time of the 3D bioprinted structures has been defined as 4D bioprinting and several works came out in the last years, which have been widely reviewed (Gao et al., 2016; Yang et al., 2020). Cardiomyocytes have the capability to self-actuate, which make them natural muscle actuators and have inspired a new generation of soft engineered devices with active motion. In fact, bioprinting have been used to fabricate bioinspired soft robots. Shin et al. (2018) created a batoid-fish inspired soft robot which was able to independently move in a swimming-like motion. In the bioink formulation, CNTs were integrated in order to give mechanical guidance and conduction capability to the GelMA substrate. Indeed, they could use the anisotropy brought from the CNTs to have an internal skeleton-like supportive structure where the cardiomyocytes could attach and locally bend a part of the substrate which generated the swimming moves (Shin et al., 2018). The construct needs to be electrically stimulated to drive the cell beating, it precludes from a wireless control of the bionic construct.

Remote control has been used to induce active motion of soft hydrogels. Kim et al. (2018) reported on the use of magnetic field for remote control of soft materials with programmed nano-magnetic domains. A printed smart soft material, with the ability to rapidly change from one to another pre-defined configuration of its shape was created. The change of configuration could take place in the same dimensional space (2D to 2D configuration) or in different dimensional spaces (2D to 3D configuration). Due to the different magnetic domain orientations, the printed structure had different responses to the external magnetic field. Therefore, it was possible to create shape-transforming structures or soft materials with moving capabilities driven by the remote control of an external magnetic field (Kim et al., 2018). In this study cells were not embedded in the soft robot and an interesting step further would be to include cells in the bioink and study their growth within a dynamic platform programmed with a set of motions. A similar approach was adopted in the work of Tognato et al. (2018) where magnetic iron oxide nano-particles (IOPs) have been used to create a biocompatible nano-composite hydrogel based on GelMA which was possible to control with the presence of a remote magnetic field. Anisotropy inside the hydrogel was created by application of a magnetic field before the crosslinking, which organized the IOPs into parallel nano-fibers oriented along the magnetic field lines. When the crosslinked GelMA with aligned IOPs was used as cell culture substrate, this material showed to induce myoblast differentiation in myotubes which were aligned along the IOPs fibers. Moreover, the nano-composite hydrogel was used as bioink for 3DBP. A star-shaped structure which could be remotely controlled in a liquid environment through an external magnetic field was fabricated (Figure 2E). In this case the swimming motion was given by discontinuous application of the magnetic field (Tognato et al., 2018). Such controllable structures are envisioned to be used, among others, in tissue engineering. Indeed, the great advantage is to have a 3D support for cells which can grow under continuous necessary stimuli and finally mature in functional tissue without directly touch them.

It is also possible to use multifunctional nano-biomaterials and create complex hydrogel constructs, which show multiple behaviors related to intrinsic characteristics of the embedded nano-particles. Augurio et al. (2019) developed a nano-composite hydrogel which could be deep monitored and remotely controlled under biological tissues. It was exploited the capability of upconverting nano-particles (UCNPs), which can convert near infrared (NIR) radiation in UV-visible emission, and the sensibility to the magnetic field of iron oxide nano-particles (IONPs) used to magnetically polarize the hydrogel. Following the application of a magnetic field, the IONPs self-assembled in nano-rods which gave magnetic anisotropy to the hydrogel matrix. Indeed, after the fabrication process the luminescent signal have been detected from the scaffold placed under ~0.6 mm layer of chicken breast muscle upon excitation at 800 nm. Also, orientation and rotation of the construct along the direction of an applied external magnetic field have been shown to be possible (Augurio et al., 2019). The excellent biocompatibility and contactless manipulation of this nano-composite hydrogels open the way toward future applications of the synergy nano-technology-bioprinting where soft-robotics devices will be fabricated for minimally invasive endoscopic tools.

## Conclusion

Synergistic combination of bioprinting and nano-technology have been demonstrated valid approach for a biofabrication process more efficient with a clear added value in TERM applications. Two main biofabrication approaches have been adopted: (a) bioprinting of cell-laden nano-composite bio-inks for direct fabrication of nano-functionalized constructs, and (b) cell-free approach where a nano-composite biomaterial is used to fabricate scaffolds. In both cases the presence of nano-features inside the biological constructs support and increase the cell differentiation toward mature tissue, but the two approaches have different benefits and applications. Nano-composite bio-inks can modify the pure hydrogel’s rheological properties. Meanwhile, the hydrogel crosslinking capability can retain nano-biomaterials inside the gel’s matrix after bioprinting with active or passive bindings. Nano-composite bio-inks have been used to trigger specific cell responses. Nevertheless, a strict classification of targeted applications is not completely clear. Overall, the nano-composite’s functionalities which were identified in this review work are related to control the biomolecule release close to the cell surrounding, to instruct cells and enhance the matrix’s properties, to trigger cellular responses and to remotely control cell behavior. Nano-featuring inside bioprinted constructs can also be achieved without directly embedding nano-biomaterials inside the bio-ink, but through the design of bio-ink compositions which can self-assemble in nano-structures (e.g., nano-fibers or nano-pores). Protein absorption, cell adhesion and differentiation have proven to be enhanced when cells were laden in self-assembled nano-featured bioinks. Moreover, nano-biomaterials can introduce passive components inside biological constructs for remotely control and contactless stimulation of the biological construct. Ultrasounds or magnetic field are useful tools to locally stimulate cells to differentiate or control the substrate movement, recreating the natural tissue’s dynamics. Bionic tissue and soft robotics are the future applications of synergistic combination of additive manufacturing and nano-technology, as they open the prospective for organ-inspired active soft tissues fabrication. Although the convergence of these two fields has been barely explored, the current results are promising milestones for the future, which will lead to a rising and synergistic use of nano and biofabrication technologies for generating hierarchically complex tissues and organs.

## Author Contributions

All authors contributed to the design and implementation of the research, analysis of the results, and writing of the manuscript.

## Conflict of Interest

The authors declare that the research was conducted in the absence of any commercial or financial relationships that could be construed as a potential conflict of interest.
